# Antifungal Mechanism of Dipicolinic Acid and Its Efficacy for the Biocontrol of Pear Valsa Canker

**DOI:** 10.3389/fmicb.2020.00958

**Published:** 2020-05-20

**Authors:** Xue-Ge Song, Ming-Hui Han, Feng He, Su-Yan Wang, Chao-Hui Li, Gui-Chun Wu, Zi-Gang Huang, Dong Liu, Feng-Quan Liu, Pedro Laborda, Xin-Chi Shi

**Affiliations:** ^1^School of Life Sciences, Nantong University, Nantong, China; ^2^College of Life Science, Anhui Normal University, Wuhu, China; ^3^Jiangsu Key Laboratory for Food Quality and Safety-State Key Laboratory Cultivation Base of Ministry of Science and Technology, Institute of Plant Protection, Jiangsu Academy of Agricultural Sciences, Nanjing, China; ^4^Jiangsu Key Laboratory of Neuroregeneration, Co-innovation Center of Neuroregeneration, Nantong University, Nantong, China

**Keywords:** Valsa canker, dipicolinic acid, fungal apoptosis, *Bacillus subtilis*, biological control

## Abstract

*Valsa pyri* is a fatal canker pathogen that causes significant reduction of crop yield in pear orchards. *V. pyri* invades the trunk phloem, and is difficult to control by chemical treatment. In this work, it was found for the first time that *Bacillus subtilis*-produced dipicolinic acid (DPA) exhibits antifungal activity against different canker pathogens, including *Alteraria alternata*, *Botryosphaeria dothidea*, *Rhizoctonia solani*, and *V. pyri*. Growth inhibition of *V. pyri* was observed at less than 5 mM concentration (pH = 5.6). DPA showed the highest antifungal activity at acidic pH values and in the presence of bivalent metals, such as zinc(II), cobalt(II), and copper(II). Measurement of mRNA expression levels and scanning electron microscope (SEM) observations revealed that DPA causes *V. pyri* apoptosis via inhibition of chitin biosynthesis and subsequent cell lysis. Interestingly, DPA showed high stability in the pear bark and was able to cross the pear tree bark into the phloem, protecting the internal phases of the pear trunk. In preventive applications, DPA reduced the canker symptoms of *V. pyri* on Cuigan pear trees by 90%. Taken together, an efficient strategy for the management of *V. pyri*-caused canker disease was developed using a novel antifungal agent, DPA, with strong antifungal activity and particular diffusion properties.

## Introduction

Pears are the third most consumed fruit after apples and grapes. Pear global production reached 24.1 million tons in 2017, with major production areas in China, Italy, the USA, Argentina and Spain (Food and Agriculture Organization of the United Nations [FAO], 2017). Pear trees are highly susceptible to a wide range of devastating pathogens. Among pear tree diseases, canker diseases are common, widespread and destructive, and result in significant economic loss and significant crop losses (Zhai et al., 2014; Chen et al., 2016). However, only few management strategies were reported until the date. In this sense, some chemical fungicides, such as thiophanate-methyl and copper (II)-humic acid complex, have been applied for the control of pear cankers in China, whereas *Lysobacter enzymogenes*-produced heat stable antifungal factor (HSAF) could reduce the symptoms of Valsa canker by 81.3% (Cheng et al., 2017). Acibenzolar-S-methyl was shown to induce systemic acquired resistance in susceptible field-grown pear trees to fire blight (Johnson and Temple, 2017).

*Valsa mali* var. *pyri*, also known as *V. pyri*, is an aggressive canker pathogen that causes pear and apple canker disease on many continents, including Asia. This fungus invades host tissue wounded by injury in the bark (He et al., 2016; Kange et al., 2020). The pathogen can invade the phloem, resulting in vascular tissue necrosis, which can result in the death of the pear tree (Yin et al., 2015; Li et al., 2016). The necrotrophic action has been attributed to secretion of pectinases and other degrading enzymes that promote the infection and colonization of the host trunk (Li et al., 2015; Yin et al., 2015). The disease is difficult to control with chemical treatments since most active compounds are not able to protect the internal phases of the tree trunk, allowing the pathogen to advance. For this reason, the most common way to contain this disease is to destroy the infected plants.

*Bacillus* species have been shown to be a promising source of metabolites with antifungal activities (Caulier et al., 2019). Among *Bacillus* species, *B. subtilis* has been thoroughly explored as biocontrol agent for the management of many plant diseases including *Valsa* canker (Santoyo et al., 2012; Liu et al., 2015; Siahmoshteh et al., 2018). *B. subtilis* E1R-J-secreted protein EP-2 showed antifungal activity against apple *Valsa* canker (Wang et al., 2016). An antifungal protein isolated from *B. subtilis* XB-1 inhibited *Monilia fructicola* growth (Ren et al., 2019; Zhou et al., 2019). *B. subtilis* C232-secreted lipopeptides inhibited microsclerotia formation in *Verticillium dahliae* (Yu et al., 2018). *B. subtilis* 7PJ-16 was used as biocontrol agent of mulberry fruit sclerotiniose (Xu et al., 2019). In this work, a new antifungal compound, dipicolinic acid (DPA), was isolated from *B. subtilis* 168 secretions, and showed strong antifungal activity against four pear canker pathogens. DPA was demonstrated to inhibit the biosynthesis of chitin in *V. pyri*, producing cell wall damage and, subsequently, the fungal death. Interestingly, DPA showed high stability in the pear bark, and was able to diffuse into the phloem, protecting the internal phases of the trunk. Preventive applications of DPA inhibited *in vivo V. pyri* growth by 90%.

## Materials and Methods

### General Information and Strains

All reagents and chemicals were used as received from commercial suppliers without further purification or modification. DPA was purchased from Macklin (China), and used in the antifungal mechanism studies. Mass spectrometry analyses were carried out in a QTRAP 5500 Linear Ion Trap Quadrupole MS/MS Mass Spectrometer (AB Sciex Instruments, United States). Fungal strains, including *A. alternata*, *B. dothidea*, *R. solani*, and *V. pyri*, were grown on potato dextrose agar medium (PDA). PDA medium was prepared by boiling 200 g of potatoes in 1 L of water for 30 min. Then, 20 g dextrose was added (pH = 5.6).

### Data Analysis

The statistical analyses were performed using SPSS (Statistical Package, Version 20.0). The variables were subjected to student’s *t*-test and were tested for significance at *P* < 0.05 (^∗^), *P* < 0.01 (^∗∗^), *P* < 0.001 (^∗∗∗^), and *P* < 0.0001 (^****^) levels (ns = no significance). The standard deviation, which was calculated using Microsoft Excel 2010, was used to quantify the dispersion.

### Media and Growth Conditions for *B. subtilis*

*B. subtilis* 168 strain was maintained on lysogeny broth (LB; 5 g yeast extract, 10 g tryptone, and 10 g sodium chloride at pH 7.0-7.2 in 1 L of distilled water) agar plate at 37°C. Seed cultures were grown at 37°C and 200 rpm in 250 mL Erlenmeyer flasks containing 50 mL LB medium until OD_600_ = 2.0. After centrifugation of 10 mL of seed culture at 8,000 × *g* and 4°C for 10 min, the collected cells were added into 100 mL LB medium for the preparation of the fermentation cultures. The fermentation cultures were shaken in a 250 mL Erlenmeyer flask at 37°C and 200 rpm for 72 h.

### Isolation and Identification of DPA

Five milliliters of *B. subtilis* 168 fermentation culture were centrifuged for 6 min at 10,000 × *g* and 4°C. Then, the filtered supernatant was studied by high-performance liquid chromatography (HPLC; Agilent 1200 series, Hewlett–Packard, United States) with an ultraviolet-visible light absorbance detector, using a high-performance carbohydrate column (250 × 4.6 mm, Waters, Japan) at 32°C, a mixture of acetonitrile/water 1:1 (pH = 3.4) as mobile phase at a constant flow rate of 0.4 mL/min (injection volume: 100 μL) for 15 min.

All the peaks in the chromatogram were collected by analytical scale HPLC. The collected peaks from 5 mL fermentation culture were evaporated using a freeze-drier, and re-dissolved in 100 μL water. The antifungal activity of the peaks was tested using *V. pyri*. To achieve this goal, the fungal pathogen was located on the center of the Petri dish containing PDA medium. Then, a 3 mm diameter hole was performed in the solid growth medium, and 30 μL of the collected peaks was poured in it. The plate was then incubated during 48 h at 28°C. The antifungal activity was detected by measurement of the diameter of the inhibition zone.

Among the collected peaks, only one peak at 10.1 min showed antifungal activity. The corresponding compound was isolated by analytical scale HPLC, and identified as DPA by mass chromatography (MS (ESI): calcd. for C_7_H_4_NO_4_ [M–H]^–^ 166.0140, found 166.0). The concentration of DPA in the fermentation culture was calculated according to the peak area. A standard curve was established from 0 to 2 mM DPA. The fermentation cultures were repeated 3 times to calculate the standard deviation.

### Antifungal Activity Assay

The antifungal activity of DPA was tested using the following plant pathogens: *A. alternata*, *B. dothidea*, *R. solani*, and *V. pyri*. Commercial DPA was used in these experiments. The antifungal screening was carried out using a similar protocol previously reported by our research group (Laborda et al., 2018). Briefly, the fungal pathogen was placed in the center of a Petri dish containing PDA medium (pH = 5.6) and DPA at 3, 5, and 10 mM, respectively. The antifungal activity was calculated by measurement of the diameter of the mycelial growth. The control treatments were performed in the absence of DPA. The dishes were incubated at 28°C for 3 days for *A. alternata*, *R. solani*, and *V. pyri*; and 7 days for *B. dothidea*. Three replicates were performed for each treatment.

### Conidium Germination Assay

*V. pyri* wild-type strain Vp297 was isolated from diseased pear trees and validated as previously reported by our research group (He et al., 2016). The isolate was preserved as a glycerol stock (20%) at -80°C in the Plant Bacteria and Biocontrol Laboratory, Institute of Plant Protection, Jiangsu Academy of Agricultural Sciences. To carry out the conidium germination assay, *V. pyri* was grown on PDA medium at 28°C for 5 days, and the mycelia were divided into 3 mm diameter plugs. Then, conidial production was induced by culturing three plugs of *V. pyri* in 40 mL barley-honey-tryptone medium at 28°C and 200 rpm for 24 h. Barley-honey-tryptone medium was prepared by heating 600 mg honey, 100 mg barley and 200 mg tryptone in 40 mL water at 121°C for 1 h (Zhao et al., 2012). Freshly harvested conidia were suspended in germination solutions (1 × 10^6^ conidia/mL), and the germination was assayed after incubation at 28°C and 200 rpm for 24 h. The germination solutions consisted of 100 μL YEPD medium (0.15 g yeast extract, 0.5 g tryptone, 1 g glucose, pH = 5, in 50 mL water) and 100 μL water with 3 mM DPA (pH = 5). Two hundred microliters YEPD/water 1:1, in the absence of DPA, was used as the control treatment. Fungal growth was detected using either a Leica DM2500 microscope at ×4, ×10, ×20, and ×40 magnifications, a Nikon A1R HD25 inverted microscope at ×4, ×10, ×20, and ×40 magnifications, or a SEM Gemini 300 instrument.

To prove whether DPA could cause cell death, the conidia were prepared as mentioned above. Then, the conidia were shaken at 28°C and 200 rpm for 24 h using a 5 mM DPA YEPD/water 1:1 solution (200 μL, pH = 5). After centrifugation of the cells, the upper phase was discarded and the cells were washed with 100 μL YEPD medium to remove the traces of DPA. Finally, the cells were suspended in 200 μL YEPD/water 1:1. The resulting suspension was stirred at 28°C and 200 rpm for 24 h, and the fungal growth was analyzed using a Leica DM2500 microscope.

The effect of pH and metals on the conidia germination was detected using 1 mM DPA. The effect of the pH was calculated at pH values 3, 4, 5, 6, 7, 8 and 9. The experiments were performed in 200 μL YEPD/water 1:1. The effect of iron(II), nickel(II), zinc(II), cobalt(II), and copper(II) on the conidia germination was examined by adding 1 mM of the corresponding metal chloride into the germination medium at pH = 5. The germination was analyzed with a Leica DM2500 microscope. The antifungal activity was calculated according to the number of conidia per μL.

### Fluorescent Live-Cell Imaging

To collect the images, the conidia were prepared as mentioned in the “Conidium Germination Assay” section. Freshly harvested conidia were suspended in a YEPD/water 1:1 solution (200 μL) containing 3 or 5 mM DPA (1 × 10^6^ conidia/mL, pH = 5). After shaking at 28°C and 200 rpm for 0, 24 or 72 h, 10 μL of the germination suspension was stained with 10 μL 4′,6-diamino-2-phenylindole (DAPI, 10 μg/mL solution, Solarbio). The stained samples were observed with an Olympus BX51 fluorescence microscope.

### Detection of mRNA Levels in *V. pyri* After Treatment With DPA

After preparation of *V. pyri* conidia following the conditions described in the “Conidium Germination Assay” section, freshly harvested conidia (5 × 10^6^ cells) were suspended in a solution containing 0.5 mL YEPD and 0.5 mL water (pH = 5). The resulting suspension was shake at 28°C and 200 rpm for 24 h. The treatment experiment was done using 3 mM DPA, whereas the control experiment was performed in the absence of DPA. The conidial suspension was centrifuged at 4°C and 10,000 × *g* for 6 min, and the cells were harvested and washed with 200 μL water. Total RNA was extracted using TRIzol reagent (Ambion, United States). The residual DNA was removed and the first-strand cDNA was synthesized in one pot using the Transcript All-in-One First-Strand DNA Synthesis SuperMix for qPCR (One Step gDNA Removal) Kit (Tsingke, China). Quantitative real-time PCR was performed using a set of 2 PCR primers with SYBR Green I Real Time PCR (Solarbio, United States). The PCR analysis was carried out using a 7500 Real time PCR system (Applied Biosystems, United States). The mRNA levels of ergosterol biosynthesis genes *VpDHCR* (*Vp_08088*), *VpHMGCR* (*Vp*_*03215*), *VpACAT* (*Vp*_*07270*), and *VpSMT* (*Vp*_*08294*), glucan biosynthesis genes *VpGS1* (*Vp*_*08744*) and *VpGS2* (*Vp*_*00561*), and chitin biosynthesis genes *VpCHS2*, *VpCHS6*, *VpCRZ1*, *VpRCR1*, and *VpRCR2*, were examined. Primers were designed according to the gene sequences using Primer Premier 5.0 (Supplementary Table S1). The DNA sequences of all studied genes were previously reported by our research group (He et al., 2016). In all cases, *VpActin* was used as a reference gene (He et al., 2018), and the relative gene expression was calculated by the 2^–Δ^ ^Δ^ ^*ct*^ method.

### Determination of Chitin Content

*V. pyri* conidia were prepared following the conditions described in the “Conidium Germination Assay” section. Freshly harvested conidia were suspended in a YEDP/water 1:1 solution (1 mL) with 3 mM DPA (pH = 5). The control experiment was performed in the absence of DPA. The resulting solution (1 × 10^6^ conidia/mL) was shake at 28°C and 200 rpm. The content of chitin was detected at 0, 1, 2 and 3 days (Kapteyn et al., 2000). Briefly, extracted cell walls were hydrolyzed in 1 mL 6 M HCl at 100°C for 17 h. After evaporation of the solvent using a freeze-drier, the samples were dissolved in 1 mL water. A 0.75 M Na_2_CO_3_ solution (100 μL) was added to 100 μL sample. The mixture was incubated at 100°C for 20 min. Then, 95% ethanol (0.7 mL) and solution A (100 μL; solution A: 1.6 g p-dimethylaminobenzaldehyde in 30 mL concentrated HCl and 30 mL ethanol) were added. The absorbance at 420 nm was measured and compared with the standard curve from 0.1 to 20 mg/mL glucosamine. The experiments were repeated 3 times.

### Detection of DPA in Pear Trunk

Two-year Cuigan pear trees in fields were covered with plastics to avoid the direct effect of the environment. Branches of approximately 2 cm diameter were selected, and sprayed with a 12 mM aqueous DPA solution (300 mL). To control the presence of DPA in the branches, one piece of pear trunk, 1 cm^2^ containing bark and phloem, was extracted from each branch with a knife. Each extracted piece was carefully divided into bark and phloem. The sample collections were carried out after 0, 1, 3, 5, 10, 15, and 20 days. Five pear branches were used for each time point, and 2 pieces of trunk were extracted from each branch (10 samples were collected for each time point). The trunk pieces were freeze-dried in Eppendorf tubes. Then, water (200 μL) was added to each tube, and the tube was stirred at 500 rpm for 15 min. The presence of DPA in the aqueous solutions was analyzed using HPLC. HPLC conditions: reversed-phase HPLC (Agilent 1200 Series, United States) at 270 nm using an Eclipse XDB-C18 column (250 × 4.6 mm, Agilent). A constant flow, 0.3 mL/min, 0.03 M H_2_SO_4_ aqueous solution was used (column temperature: 60°C; injection volume: 50 μL). DPA appeared at 6.7 min retention time using the aforementioned conditions. The concentration of DPA in the solutions was calculated according to the peak area using a standard curve from 0 to 10 mM DPA.

### Curative and Preventive Efficacies of DPA for the Management of Valsa Canker

To evaluate the preventive and curative efficacies of DPA, *V. pyri* conidia was produced following the conditions described in the “Conidium Germination Assay” section. For the preventive assay, 2 mm diameter holes were dug on branches of 2-year Cuigan pear trees. Then, a DPA aqueous solution (12 mM, 300 mL, pH = 5) was sprayed on 6 branches, whereas water (300 mL, pH = 5) was sprayed on another 6 branches as a control treatment. After 2 h, 10 μL conidial suspension (1 × 10^9^ conidia/mL) was injected into each hole. Sixty inoculation sites were used in the study (30 inoculation sites for the treatment group, and 30 inoculation sites for the control group). Five inoculation points were carried out in each branch. After 7 days, the preventive efficacy was measured according to the lesion length. For the curative assay, sixty 2-mm-diameter holes were dug on the branches of 2-year Cuigan pear trees (12 branches were used with 5 holes in each one), and 10 μL conidial suspension (1 × 10^9^ conidia/mL) was injected into each hole. After 48 h, a DPA aqueous solution (12 mM, 300 mL, pH = 5) was sprayed on 6 branches, whereas water (300 mL, pH = 5) was sprayed on the other 6 branches as a control experiment. After 6 days, the curative efficacy was measured according to the lesion length. Pear tree branches of approximately 2 cm diameter were used in these experiments. *V. pyri* was successfully inoculated in all the inoculation sites, and the symptoms were easily observed after removing the bark with a knife. All results were used to calculate the average lesion length and deviation.

## Results

### Antifungal DPA Was Identified in *B. subtilis* 168 Secretions

**FIGURE 1:**
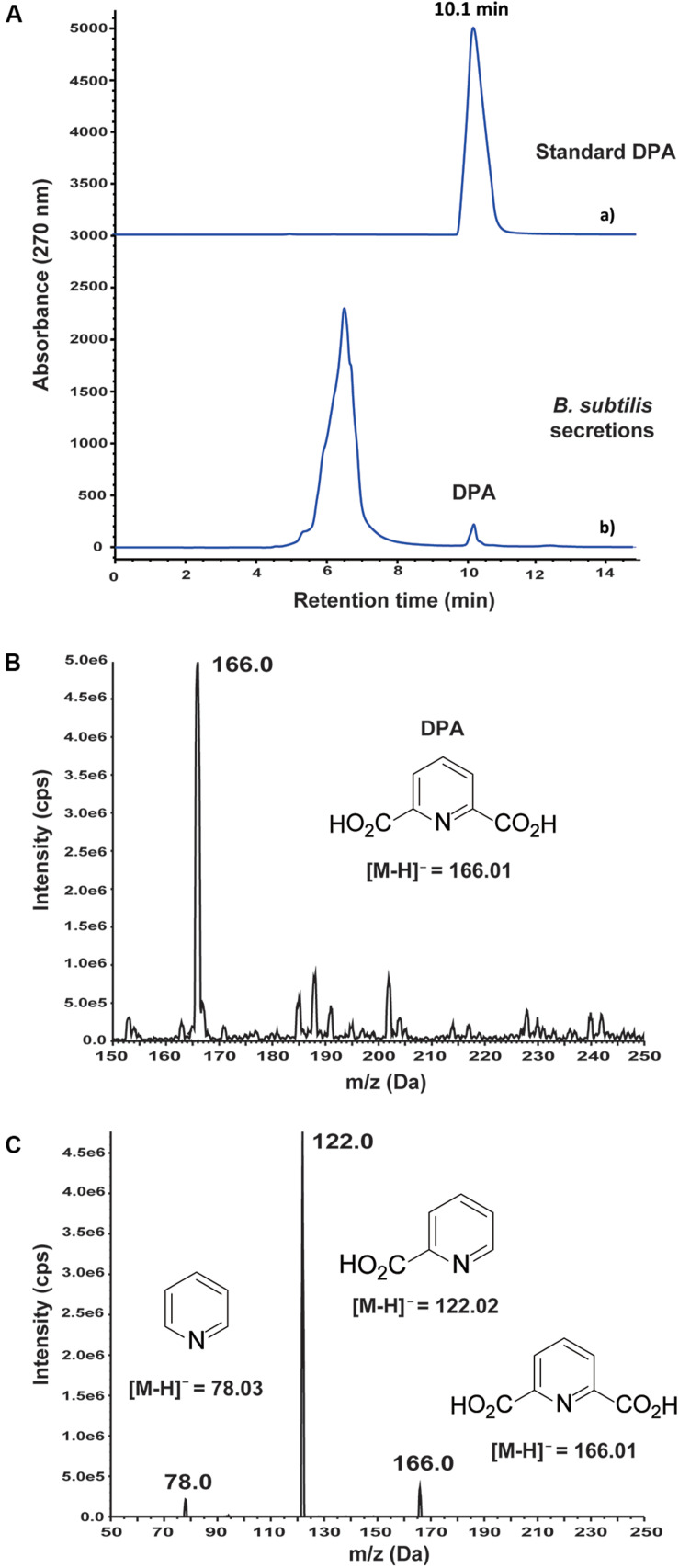
Detection and identification of dipicolinic acid (DPA) in the secretions of wild-type *Bacillus subtilis* 168. **(A)** (a) Standard DPA; (b) HPLC-based study of *B. subtilis* 168 secreted metabolites. DPA was detected at 0.24 ± 0.01 mM concentration after growing *B. subtilis* 168 for 72 h in LB medium. **(B)** MS analysis of *B. subtilis* 168-secreted DPA. **(C)** MS/MS analysis of the m/z peak at 166.0 Da.

An antifungal metabolite was detected by HPLC at 10.1 min retention time in the secretions of wild-type *B. subtilis* 168 (Figure 1A). After purification of the active compound by HPLC, it was analyzed by mass spectrometry. MS analysis in negative mode revealed a main m/z peak at 166.0 (Figure 1B), which is consistent with the expected molecular weight of a pyridine dicarboxylic acid structure, [M–H]^–^ = 166.0140. MS/MS-analysis of this m/z peak revealed ions at 121.9 and 78.0 (Figure 1C), demonstrating the presence of a pyridine dicarboxylic acid. Two different metabolites based on pyridine dicarboxylic acid structures, pyridine-2,3-dicarboxylic acid and DPA, are common metabolites in *Bacillus* secretions. It must be noted that DPA contains the carboxylic acid groups at positions 2 and 6 of the pyridine ring. In order to discern between both possible structures, pyridine-2,3-dicarboxylic acid and DPA were purchased from commercial suppliers and studied by HPLC. Pyridine-2,3-dicarboxylic acid appeared at 13.2 min retention time in the HPLC spectrum (Supplementary Figure S1), whereas the retention time of DPA was in well agreement with the observed antifungal metabolite. The concentration of DPA in the secretions of *B. subtilis* 168 was 0.24 ± 0.01 mM after 72 h cultivation in LB medium.

### DPA Caused the Apoptosis of *V. pyri*

**FIGURE 2:**
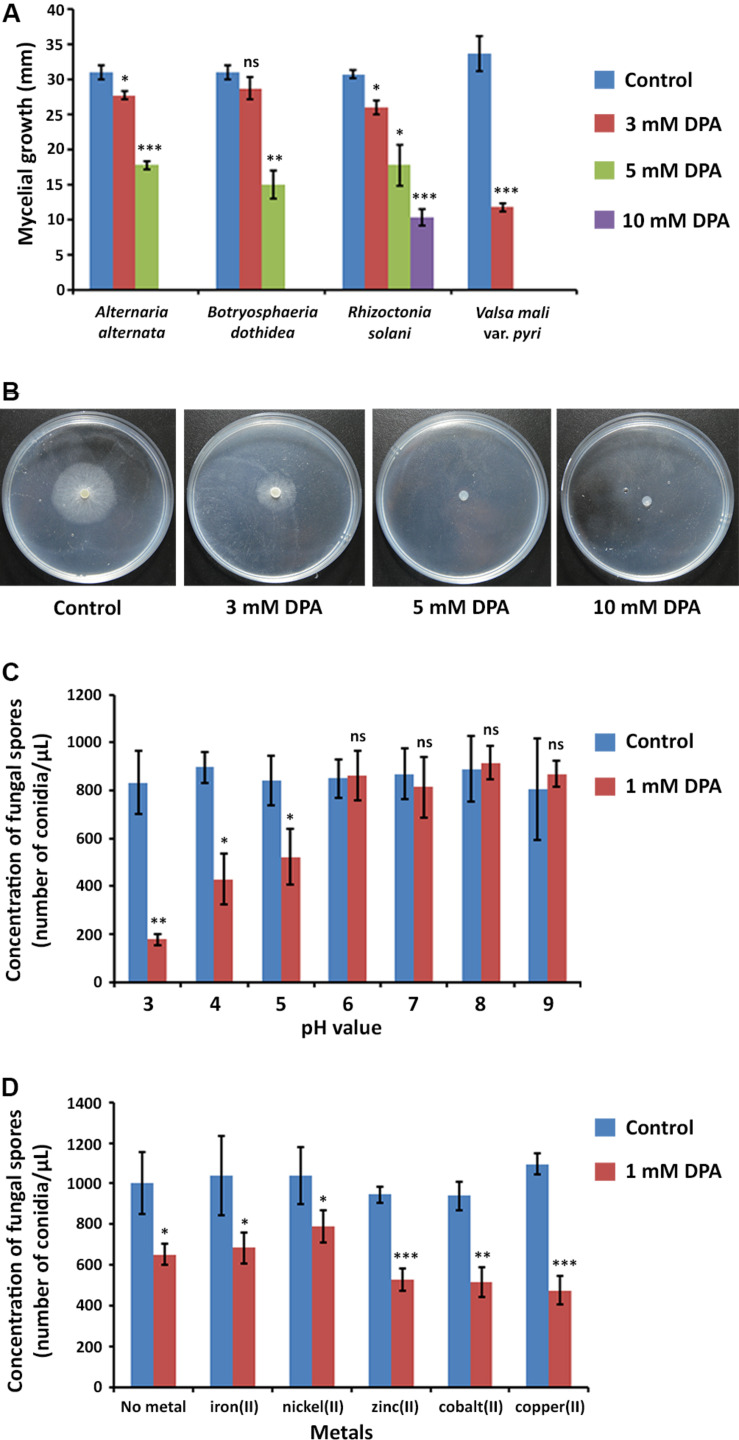
Antifungal properties of DPA. **(A)** Antifungal activity of DPA against canker pathogens *A. alternata*, *B. dothidea*, *R. solani*, and *V. pyri*. The antifungal activity was calculated according to the mycelial growth of the fungi (blue color) and the mycelial growth of the fungi in the presence of 3 mM (red color), 5 mM (green color) and 10 mM (purple color) DPA. **(B)** Mycelial growth of *V. pyri* in the presence of 0, 3, 5, and 10 mM DPA. DPA could block the growth of *V. pyri* at 5 mM concentration (pH = 5.6). **(C)** pH-dependence of DPA (1 mM) antifungal activity. The antifungal activity was calculated according to the number of conidia/μL. DPA showed higher antifungal activity at acid pH values. **(D)** Effect of metals at 1 mM concentration on the antifungal activity of 1 mM DPA (pH = 5). The antifungal activity was calculated according to the number of conidia/μL. Zinc(II), cobalt(II) and copper(II) enhanced the antifungal activity. Significance levels at *P < 0.05, **P < 0.01, ***P < 0.001, and no significance (ns).

The inhibitory activity of DPA was measured according to the mycelial growth of the fungal pathogens on PDA medium at pH = 5.6 (Figures 2A,B). DPA was able to block completely the growth of the *V. pyri* at 5 mM concentration, whereas the complete inhibition of *B. dothidea* and *A. alternata* was detected at 10 mM DPA (Supplementary Figure S2). The growth of *R. solani* was only inhibited by 70% at 10 mM DPA.

**FIGURE 3:**
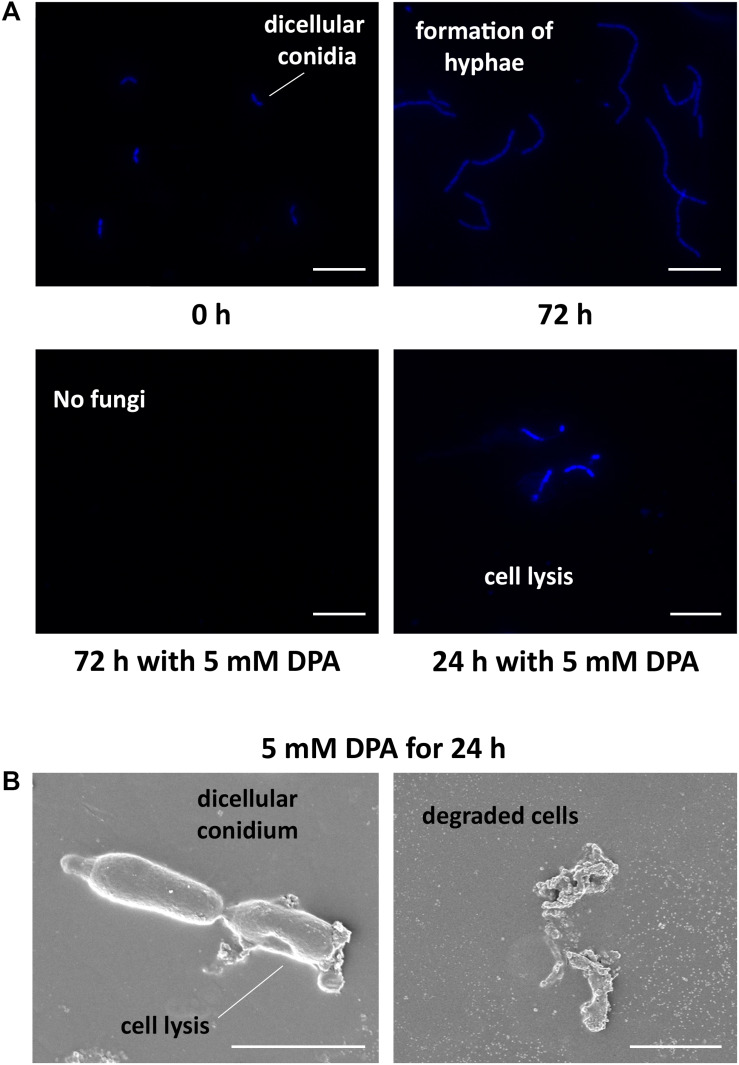
Microscope observations of *V. pyri* cell cycle and antifungal effects of DPA. **(A)** Observations of DAPI-stained *V. pyri* dicellular conidia and hyphae formation using a fluorescent microscope. The treatment of *V. pyri* conidia with 5 mM DPA (pH = 5) caused the apoptosis of *V. pyri*. Scale bar = 10 μm. **(B)** Observations of the effects of 5 mM DPA on *V. pyri* cell wall using scanning electron microscope (SEM). The treatment was carried out for 24 h at pH = 5. DPA caused the distorsion of *V. pyri* cell wall, producing the cell lysis. Scale bar = 2 μm.

The obtained results encouraged us to study the antifungal properties of DPA using *V. pyri* conidia. *V. pyri* conidia could not grow after treatment with 5 mM DPA for 24 h at pH = 5, indicating that DPA is able to cause irreversible effects on the fungi and to produce the cell death. Observations using microscope revealed for the first time that *V. pyri* forms mainly 4 μm long dicellular conidia, which showed a “banana-like” shape (Figure 3A). It must be noted that tetracellular, tricellular and unicellular conidia were also detected in low proportion. Although DAPI commonly allows the observation of the fungal nuclei, *V. pyri* nuclei were not observed. However, the cell wall and septa separations of *V. pyri* were clearly recognized after the DAPI stain (Supplementary Figure S3). In favorable conditions for 72 h, *V. pyri* conidia germinated and formed multicellular structures. However, the treatment of *V. pyri* conidia with 3 mM DPA reduced the number of conidia, suggesting that DPA is producing the cell lysis. It was observed that the treatment of *V. pyri* conidia with 5 mM DPA for 72 h resulted in no observable cells after DAPI stain. Although the presence of 3 mM DPA reduced the number of conidia, some conidia could germinate and form small multicellular structures.

The formation of small amounts of *V. pyri* conidia has been traditionally achieved by growing the fungi on PDA medium. However, the methodology reported by Zhao et al. (2012) for the production of *V. ceratosperma* conidia based on the incubation of the fungi with barley and honey was used in this occasion, and allowed the production of large amounts of *V. pyri* conidia.

*V. pyri* conidia could grow at different pH values, from 3 to 9, without relevant changes in growth or number of conidia (Figure 2C). It was found that the antifungal activity of DPA strongly depended on the pH, obtaining the highest antifungal activities at acid pH values. In this sense, the number of conidia decreased by 79% at pH = 3 in the presence of 1 mM DPA (Supplementary Figure S4), whereas the number of conidia was 52 and 38% lower when using pH 4 and 5, respectively. No antifungal effect was detected with 1 mM DPA at pH higher than 5.

DPA has been reported to be able to link to metals forming metallic complexes (Abdolmaleki et al., 2018). Here, the antifungal activity of 1 mM DPA in the presence of different metals, including iron(II), nickel(II), zinc(II), cobalt(II) and copper(II), was studied using *V. pyri* conidia at pH = 5 (Figure 2D). The obtained results indicated that zinc(II), cobalt(II) and copper(II) at 1 mM concentration can enhance the antifungal activity of DPA. The highest antifungal activity was detected in the presence of copper(II), which reduced the number of conidia by 54%, whereas the number of conidia was reduced by only 35% in the absence of metals. On the other hand, no significant changes in the antifungal activity were observed when using iron(II) and nickel(II).

### DPA Inhibits Chitin Biosynthesis in *V. pyri*

**FIGURE 4:**
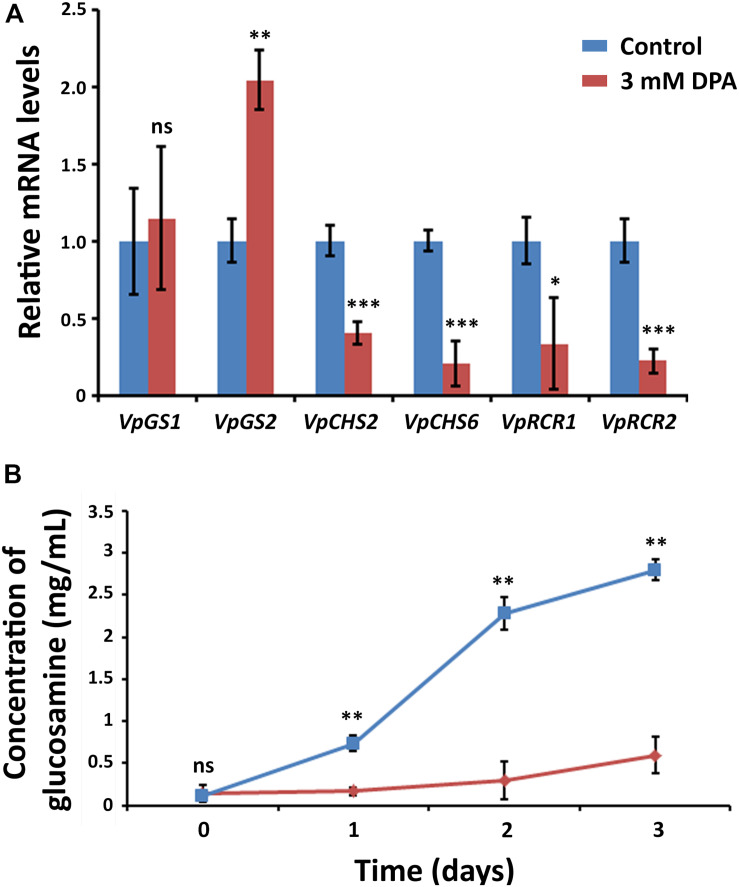
Metabolic effects of DPA. **(A)** Measurement of the mRNA levels in DPA-treated *V. pyri* conidia. Glucan biosynthesis genes *VpGS1* and *VpGS2* and chitin biosynthesis genes *VpCHS2*, *VpCHS6*, *VpRCR1*, and *VpRCR2* were examined. *V. pyri* was treated with 3 mM DPA (pH = 5), whereas the control experiments were performed in the absence of DPA. The obtained results indicated that DPA is inhibiting the biosynthesis of chitin. **(B)** Determination of chitin concentration in *V. pyri* after treatment with 3 mM DPA at pH = 5. The control experiment was carried out in the absence of DPA. The concentration of chitin was lower in the DPA-treated fungi in comparison to that in the non-treated fungi, demonstrating that DPA is inhibiting the biosynthesis of chitin. Significance levels at *P < 0.05, **P < 0.01, ***P < 0.001, and no significance (ns).

In order to clarify the antifungal mechanism, DPA-treated *V. pyri* conidia was studied using SEM and inverted microscope (Figure 3B and Supplementary Figure S5). It was found that the cells in the absence of DPA showed a round morphology. In contrast, the treatment with 5 mM DPA for 24 h produced holes in the fungal membrane, causing the apoptosis. The partial degradation of the cell wall was detected in some occasions, whereas the complete degradation of the cell wall integrity was found in other conidia. This last option resulted in the appearance of an amorphous residue, which was easily observed using SEM and inverted microscope. These results suggested that DPA is causing the distortion of the cell wall integrity and, thus, producing the cell lysis. Three different kinds of antifungal agents based on the alteration of the cell wall integrity were reported: ergosterol, glucan and chitin biosynthesis inhibitors (Sharon et al., 2009). In order to discern between the 3 possibilities, the mRNA expression levels of relevant genes involved in the 3 processes were studied in the presence of 3 mM DPA. Ergosterol biosynthetic pathway involves *VpDHCR*, *VpHMGCR*, *VpACAT*, and *VpSMT* genes, which encode *N*-acetylglucosamine-phosphate mutase, 3-hydroxy-3-methylglutaryl-CoA reductase, sterol *O*-acyltransferase and *S*-adenosyl-methionine-sterol-C- methyltransferase, respectively. Although ergosterol biosynthesis inhibitors have been demonstrated to kill fungal cells by forming pores in the plasma membrane (Sharon et al., 2009), there were no significant difference between the expression levels of the ergosterol biosynthesis genes in the treated and non-treated cells, suggesting that DPA antifungal mechanism is not related to the ergosterol biosynthesis (Supplementary Figure S6). *VpGS1* and *VpGS2* encode α-1,3-glucan synthases, which participate in the synthesis of the cell wall polysaccharides. It must be noted that no β-1,3-glucan synthase was identified in *V. pyri* until date. The mRNA level of *VpGS1* in the treated cells was similar to that in the non-treated cells, whereas the mRNA level of *VpGS2* in the treated cells was 1.79-fold higher than that in the non-treated cells, indicating that DPA is inducing the overexpression of *VpGS2* (Figure 4A). The chitin synthase genes *VpCHS2* and *VpCHS6* were 2.5- and 4.9-fold, respectively, downregulated in the treated fungi. In agreement with this result, the expression levels of *VpRCR1* and *VpRCR2*, which are responsible for the deposition of chitin on the cell wall, were 3 and 4.5 times lower in the treated cells than those in the non-treated cells (Figure 4A). These results suggest that DPA is inhibiting the biosynthesis of chitin in *V. pyri*. Although our research group reported that transcription factor *VpCRZ1* is involved in the regulation of chitin biosynthesis (He et al., 2016), no significant difference was observed in the mRNA level of *VpCRZ1* in the DPA-treated cells, indicating that DPA is inhibiting chitin biosynthesis in a *VpCRZ1*-independent manner. In order to confirm that DPA is inhibiting chitin biosynthesis, the concentration of chitin in the DPA-treated cells was measured at 0, 1, 2, and 3 days (Figure 4B). In agreement with the mRNA levels, the concentration of chitin in the fungi only slightly increased in the presence of 3 mM DPA. In contrast, the concentration of chitin in the fungi without DPA greatly increased over time, achieving the maximum after 3 days.

### DPA Is Able to Diffuse in the Pear Bark Into the Phloem and Inhibits the Growth of *V. pyri* in Pear Trunk

**FIGURE 5:**
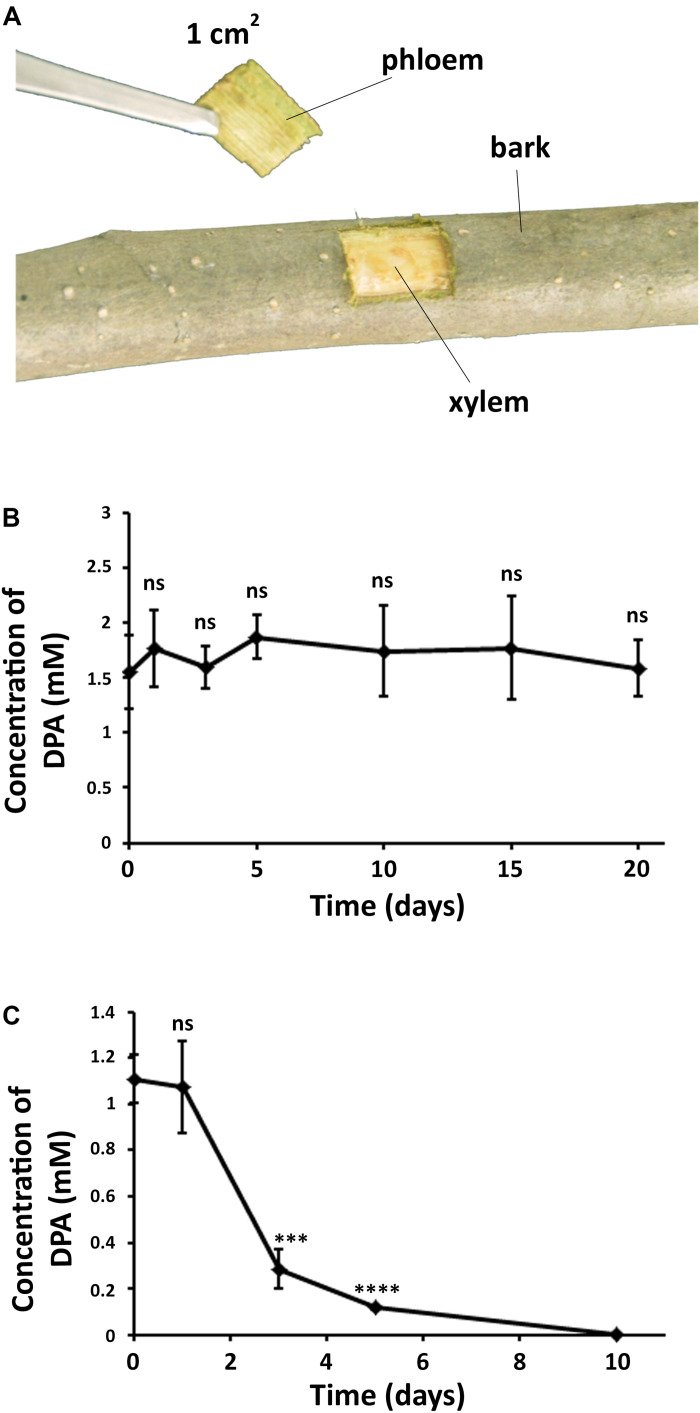
Diffusion and stability of DPA in the pear trunk. **(A)** Extraction of samples from DPA-treated branches in pear trees. DPA treatment was carried out by spraying a 12 mM DPA solution (300 mL) on the pear branches. **(B)** Concentration of DPA in pear bark over time. **(C)** Concentration of DPA in pear phloem over time. The obtained results indicated that DPA can cross the pear bark and shows high stability in the pear bark. Ten samples were collected for each time point. Significance levels at ***P < 0.001, ****P < 0.0001, and no significance (ns).

DPA was found to be present in both bark and phloem of pear trees after the treatment (Figure 5 and Supplementary Figure S7), demonstrating that DPA is able to cross the tree bark into phloem. It must be noted that no DPA was detected in the xylem, which suggests that the diffusion of DPA into deeper trunk phases is not occurring. The concentration of DPA remained unaltered in the bark for 20 days, indicating that DPA shows high stability in the pear bark. However, DPA significantly degraded in the phloem at 3 days after the treatment, and no DPA could be detected after 10 days. The concentration of DPA in the phloem remained stable during the first 24 h.

**FIGURE 6:**
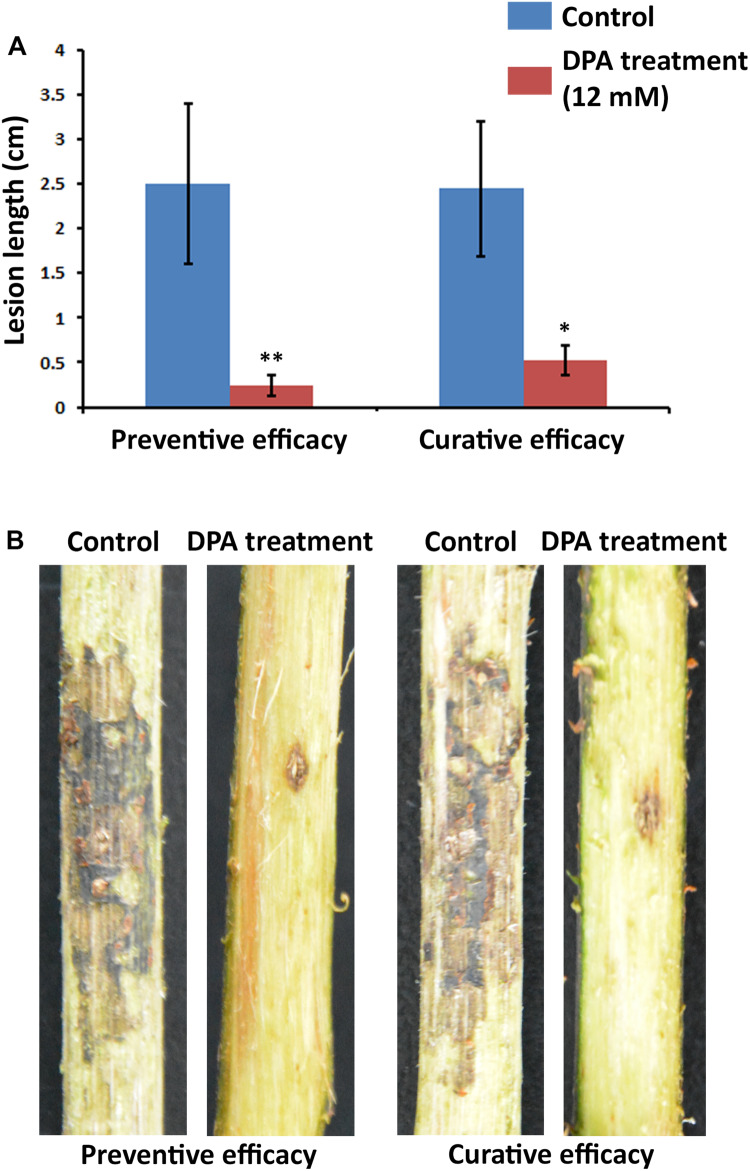
Symptoms of Valsa canker, caused by *V. pyri*, in wounds of Cuigan pear trees. **(A)** Canker lesion length after inoculation of *V. pyri* conidia. DPA treatment was performed by spraying a DPA aqueous solution (12 mM). Water was sprayed in the control experiment. **(B)** Images of the symptoms of Valsa canker after application of DPA (12 mM). Both curative and preventive efficacies of DPA were examined. The obtained results indicated that DPA can reduce the symptoms of Valsa canker, achieving the highest inhibitory activity in preventive applications. Thirty inoculation sites were studied for each condition. Significance levels at *P < 0.05, and **P < 0.01.

The curative and preventive abilities of DPA to reduce the symptoms of *V. pyri* in wounds of pear trunks were examined. To achieve this goal, *V. pyri* conidia was used in the inoculations. The disease advancement in the trunk was measured according the produced lesion length in 2-year Cuigan pear trees (Figure 6 and Supplementary Figure S8). Interestingly, DPA at 12 mM concentration could reduce the lesion length by 79% in curative applications; whereas, in preventive applications, DPA reduced the lesion length by 90%. Thus, the obtained results demonstrated that DPA is an efficient agent for the biocontrol of Valsa canker.

## Discussion

The biosynthetic pathway of DPA in *B. subtilis* is well known, and consists of the dihydropicolinic acid synthase-catalyzed condensation of pyruvate and L-aspartyl-β-semialdehyde to give 2,3-dihydrodipicolinic acid, which is then transformed into DPA via DPA synthetase (Chasin and Szulmajster, 1967). The DPA synthetase is encoded by the *spoVF* operon. DPA has been mainly related to the formation of *Bacillus* spores since DPA composes from 5 to 15% of the dry weight of the spores (Rao et al., 2016; Barak, 2017). It must be remarked that, although DPA is a common metabolite in *B. subtilis* strains, the antifungal activity of DPA was not reported in previous studies.

Although the detected concentration of DPA in wild-type *B. subtilis* 168 secretions (0.24 mM) was lower than the minimal inhibitory concentration (5 mM), previous reports have demonstrated that recombinant *B. subtilis* 168 strains can produce high amounts of DPA (>5 mM), indicating that *B. subtilis* 168 is an interesting tool for DPA production. In this sense, the replacement of the *spoVFA* promoter with another highly expressed promoter, *spoVG*, in *B. subtilis* vegetative cells, together with improving the medium composition, increased DPA production up to 170 mM (Takahashi et al., 2015). Toya et al. (2015) reported that the replacement of the *spoVFA* promoter and simultaneous deletion of acetoin synthesis genes (*alsSD*) led to 30 mM DPA after 40 h of fermentation in synthetic medium. In contrast with our results, Takahashi et al. (2015) indicated that DPA was not detected in the culture medium when using wild-type *B. subtilis* 168. Although the detection limit of the analysis method was not described, the lowest DPA concentration reported in that article was 1.26 mM when using recombinant strains. Here, DPA was found at lower concentrations using wild-type *B. subtilis* 168, indicating that the wild-type bacteria is also able to produce small amounts of DPA when using standard fermentation conditions.

DPA showed strong antifungal activity against hazardous canker pathogens, including *A. alternata*, *B. dothidea*, *R. solani*, and *V. pyri*. *A. alternata* was reported to cause stem canker in tomato plants, whereas potato stem canker is caused by *R. solani* (Tsror, 2010; Shao et al., 2019). *B. dothidea* and *V. pyri* are able to produce canker disease in apple and pear trees (Zhai et al., 2014; Yin et al., 2015). The obtained results indicated that DPA is producing *V. pyri* apoptosis via inhibition of chitin biosynthesis. In filamentous fungi (such as *V. pyri*), chitin comprises up to 15% of the cell wall mass. Chitin is connected by covalent and ion bonds to other polysaccharides, pigments and proteins, conferring rigidity. As observed in DPA-treated *V. pyri*, low chitin concentrations have been reported to induce osmotic cell lysis in different fungal species, such as *S. cerevisiae* or *Aspergillus nidulans* (Takeshita et al., 2006; Luu et al., 2019). Several antifungal agents are known to block fungal growth via inhibition of chitin biosynthesis, and this fact has been used to combat fungal pathogens in agriculture and forestry (Merzendorfer, 2013). For example, chitin biosynthesis inhibitor polyoxin D is commercially available and has been extensively used for the management of rice sheath blight in Japan (Merzendorfer, 2013). Cyclopentene-1,3-dione was identified in *Acca sellowiana* and was shown to inhibit chitin biosynthesis in *Candida* spp. (Mokhtari et al., 2018). Phenazine-1-carboximade inhibited the mycelial growth of *R. solani* by inhibiting the activity of the chitin synthases (Yu et al., 2018).

The inhibition of the β-1,3-glucan synthases in *A. infectoria* using caspofungin resulted in the upregulation of the chitin synthases as a compensatory effect (Fernandes et al., 2014). The compensatory effect between chitin and glucan synthases was also observed in *Candida albicans* and *Aspergillus fumigatus* (Fortwendel et al., 2009). The obtained results indicated that DPA is inducing the upregulation of *VpGS2* and, at the same time, the downregulation of chitin synthases and chitin deposition genes. The upregulation of *VpGS2* must be produced in *V. pyri* as a compensatory effect of chitin biosynthesis inhibition.

Our research group has recently reported the sorption of antifungal *p*-aminobenzoic acid (pABA) into the internal phases of pear fruits (Laborda et al., 2018, 2019). In that work, pABA inhibited the symptoms of *Colletotrichum fructicola*, and started to degrade in the pear skin and mesocarp at 5 days after the treatment. The diffusion ability of some organic compounds in fruits has been related to their low molecular weight (Baur and Schönherr, 1995). Similarly, some organic toxins, such as trichloroethylene (MW = 131.40), tetrachloroethylene (MW = 165.83), pyrene (MW = 202.25), and hexachlorobenzene (MW = 284.80), can be absorbed by pear barks, and this ability has been also related to their low MW (Li et al., 2005, 2010; Gopalakrishnan et al., 2009). As mentioned in the introduction section, *V. pyri* invades the pear phloem and, for this reason, *V. pyri* canker is difficult to control with chemical agents. Here, we explored the diffusion ability of antifungal DPA, which shows a low molecular weight (MW = 167.02), in the trunk of pear trees, demonstrating that DPA can be absorbed by both pear bark and, interestingly, phloem. Previous reports indicated that fungicides with half lifes (T_1__/__2_) of 5–7 days are suitable to control pear and apple diseases (Bhat et al., 2015). Our results indicated that DPA was stable in the pear bark for more than 20 days, which demonstrates that DPA is a suitable antifungal agent for the management of trunk diseases. Although DPA was shown to be less stable as in the phloem than in the bark, no degradation could be detected during the first 24 h. As previously indicated, DPA was able to cause the death of *V. pyri* in only 24 h, which suggests that this dissipation time must be enough to control Valsa canker. As far as we know, this is the first time that the diffusion ability of an antifungal agent is studied in tree trunks.

In agreement with the observed antifungal properties and diffusion ability, DPA reduced the symptoms of *V. pyri* in pear trees, obtaining the highest inhibitory activity in preventive applications. The developed strategy supposes one of the first efficient chemical methodologies for the management of Valsa canker.

## Data Availability Statement

The datasets supporting the conclusions of this article are included within the article and its additional files.

## Author Contributions

PL, X-CS, and F-QL designed the experiments. X-GS, M-HH, DL, Z-GH, and S-YW performed the experiments. FH, C-HL, and G-CW analyzed the data. PL and X-CS drafted the manuscript. All authors read and approved the final manuscript.

## Conflict of Interest

The authors declare that the research was conducted in the absence of any commercial or financial relationships that could be construed as a potential conflict of interest.

## Abbreviations

*ACAT*: acyl-CoA sterol acyltransferase
*B. subtilis*: *Bacillus subtilis*
*CHS*: chitin synthase gene
*CRZ*: Ca^2+^/calcineurin-dependent transcription factor
DAPI: 4′,6-diamino-2-phenylindole
*DHCR*: **7-**dehydrocholesterol reductase
DPA: dipicolinic acid
ESI: electrospray ionization
FAO: Food and Agriculture Organization of the United Nations
*GS*: glucan synthase gene
h: hours
*HMGCR*: 3-hydroxy-3-methylglutamyl-CoA reductase
HPLC: high-performance liquid chromatography
LB: lysogeny broth
min: minutes
mRNA: messenger RNA
MS: mass spectrometry
ns: no significance
MW: molecular weight
pABA: *p*-aminobenzoic acid
PDA: potato dextrose agar
qPCR: real-time polymerase chain reaction
*RCR*: congo red resistance gene
rpm: revolutions per minute
r.t.: room temperature
*SMT*: sterol C24-methyl transferase gene
YEPD: yeast extract peptone dextrose
*V. pyri*: *Valsa mali* var. *pyri*.
