# Supplementary material for: Antifungal Mechanism of Dipicolinic Acid and Its Efficacy for the Biocontrol of Pear Valsa Canker
Source: Front Microbiol. 2020 May 20;11:958. doi: 10.3389/fmicb.2020.00958 (PMC7251846; doi:10.3389/fmicb.2020.00958)
Supplement: Supplementary file 1 [file Data_Sheet_1.pdf]

Supporting Information

Antifungal Mechanism of Dipicolinic Acid and Its Efficacy for the Biocontrol of Pear Valsa Canker

Xue-Ge Song,[#,1] Ming-Hui Han,[#,1] Feng He,[2] Su-Yan Wang,[1] Chao-Hui Li,[3] Gui-Chun Wu,[3] Zi-Gang Huang,[1,4] Dong Liu,[1,4] Feng-Quan Liu,[3,*] Pedro Laborda[1,*] and Xin-Chi Shi[1,*]

[1]School of Life Sciences, Nantong University, Nantong 226019, People's Republic of China

[2]College of Life Science, Anhui Normal University, Wuhu 241000, People's Republic of China

[3]Institute of Plant Protection, Jiangsu Academy of Agricultural Sciences, Jiangsu Key Laboratory for Food Quality and Safety-State Key Laboratory Cultivation Base of Ministry of Science and Technology, Nanjing 210014, People's Republic of China

[4]Co-innovation Center of Neuroregeneration, Jiangsu Key Laboratory of Neuroregeneration, Nantong University, Nantong 226001, People's Republic of China

[#]These authors contributed equally to this work.

*Corresponding authors:

Dr. Xin-Chi Shi; email: shxch0301@ntu.edu.cn

Prof. Pedro Laborda; email: pedro@ntu.edu.cn

Prof. Feng-Quan Liu; email: fqliu20011@sina.com

Table S1. Primers used in RT-qPCR.

Gene	Primer_F (5´-3´)	Primer_R (5´-3´)
VpActin	AGCGTGGTATCCTGACTCTGC	CTCGGTTGGACTTGGGGTT
VpACAT	CGCAAACCTGTCCACTCCCA	AGTCCCGTCTGTTCACCCGC
VpCHS2	TGGTCGCAGCCCAAAACT	GAGAAGGCACCTGGCAACAC
VpCHS6	TGCCAGACGAAGAGCAGAAGA	GCAGCGGATGAATGGAAGAG
VpCRZ1	TCAATACGCCGCTCTTCG	GGTGCCTGTAGGCCATAGT
VpDHCR	GACTGACACCTGACACGGTT	GAGGTCAGCGAGACGGGTTA
VpGS1	TTTGGGTATGGACGGTAGCA	TGATTGCTGAAGAACTTGCGAG
VpGS2	TCCGTGTACCGTTTCCTGAC	CTCTACCACGTCAGTTTGCT
VpHMGCR	TACCCCTCTATTCACTACGG	CAACCTTCACAAGACTTCTCG
VpRCR1	GAATAAACGCGAAATGGATGC	ACCACCAGTTGACCTGAGCC
VpRCR2	AGAAGGAGGTTGCGAAAGGG	GTGTTGTTACCGGGTGGAGG
VpSMT	AGTCGCTCCAGTGAGACCCT	CTGTAACGACGACGGTTAAG

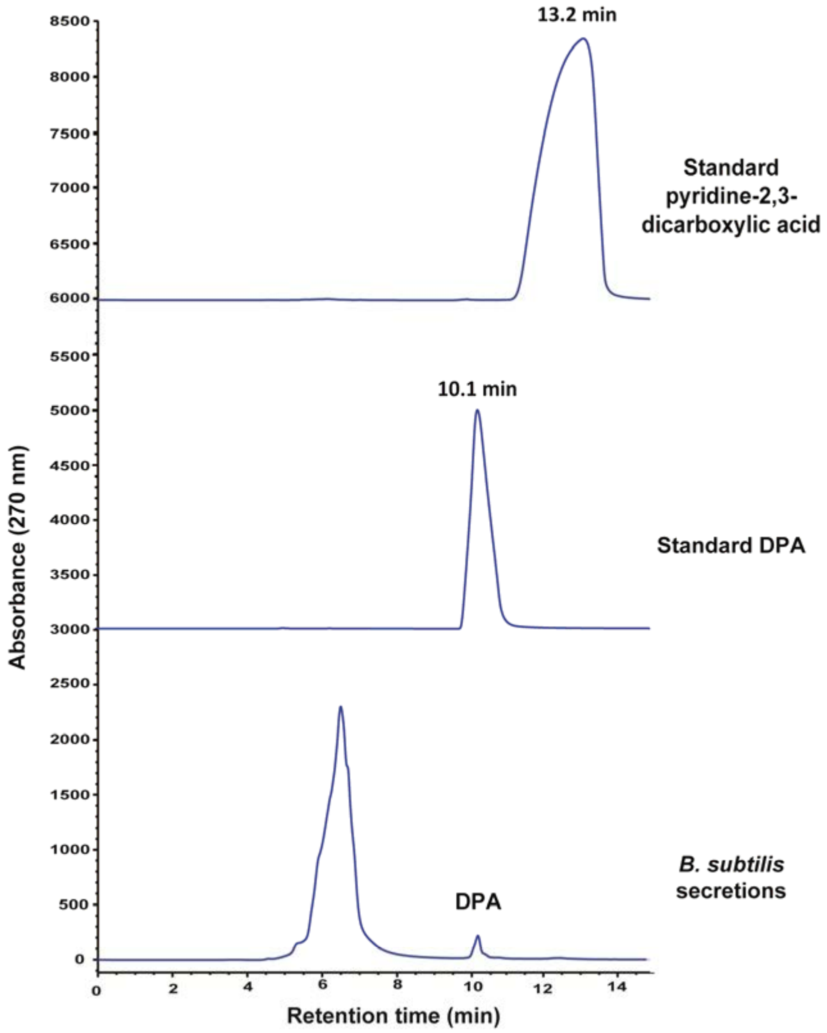

Figure S1. HPLC-based analysis of ***Bacillus subtilis*** 168 secretions. DPA appeared at 10.1 min retention time. Pyridine-2,3-dicarboxylic acid was not detected in the secretions of ***B. subtilis*** 168.

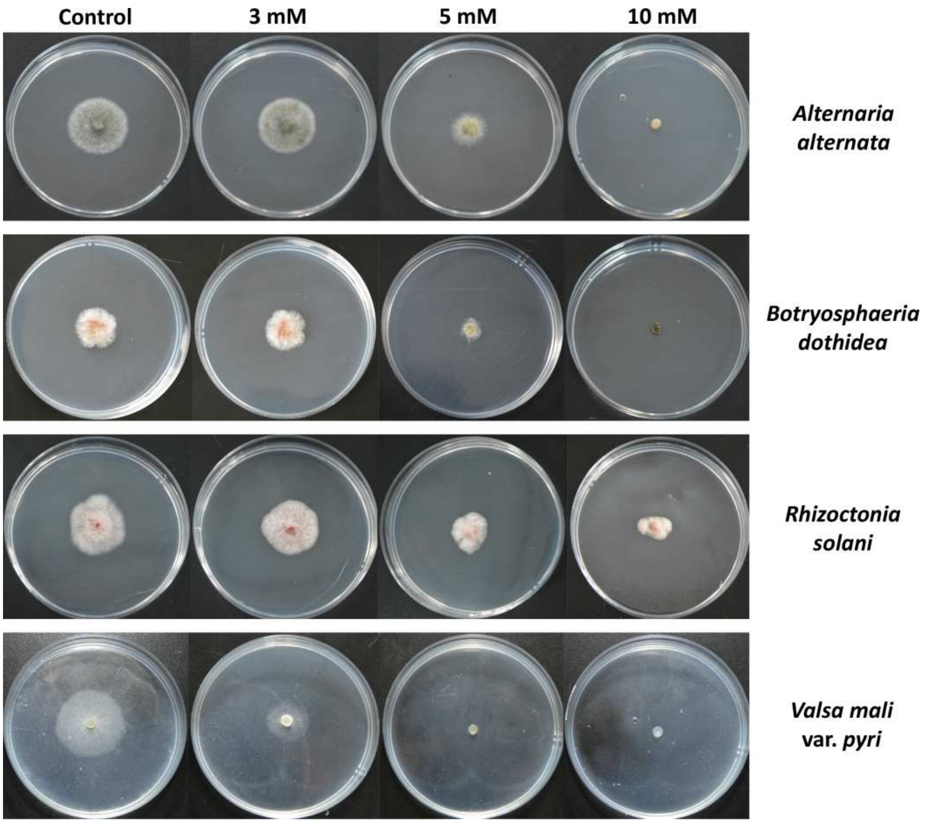

Figure S2. Mycelial growth of ***A. alternata***, ***B. dothidea***, ***R. solani*** and ***V. pyri*** in the presence of 0, 3, 5 and 10 mM DPA.

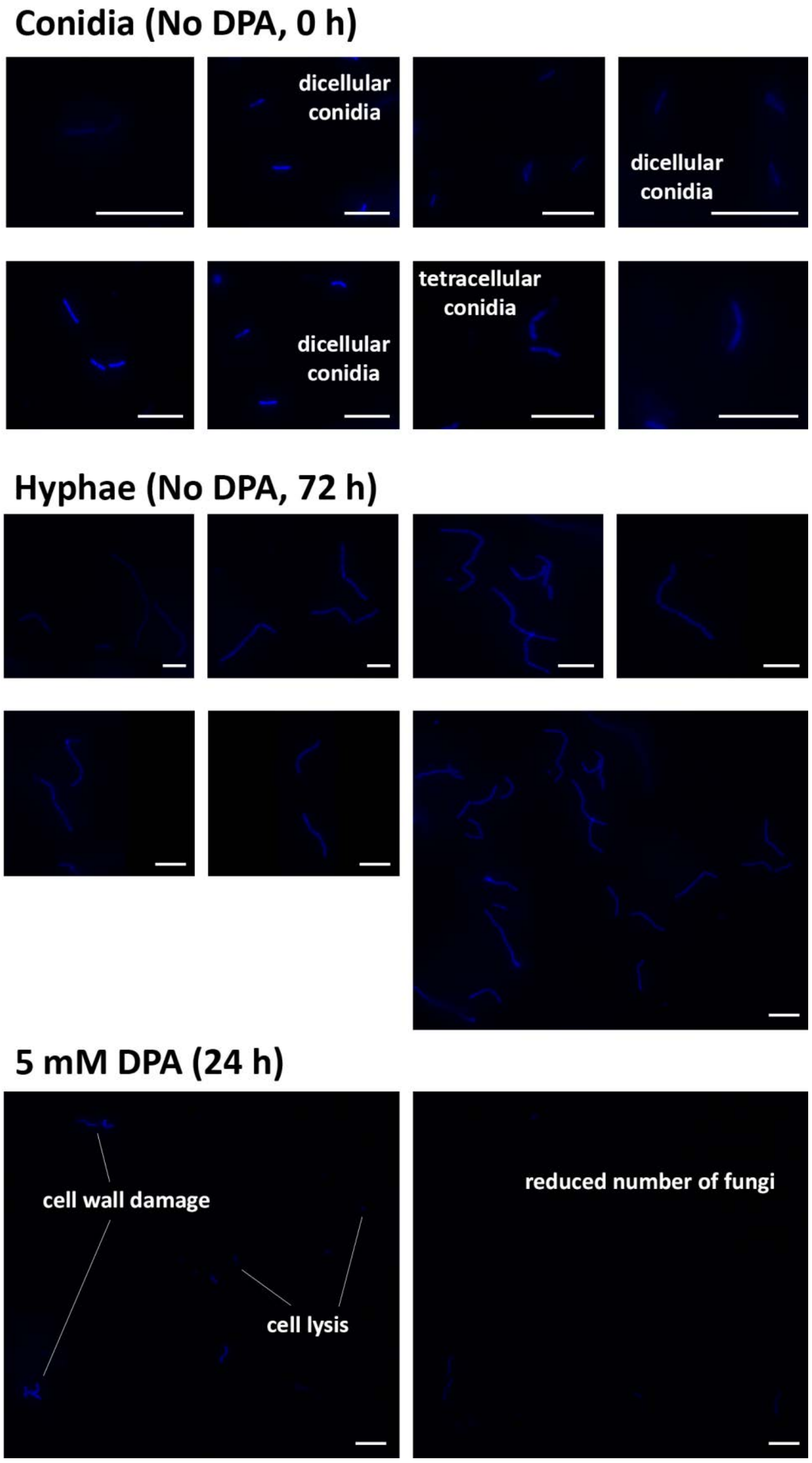

Figure S3. Observations of DAPI-stained ***V. pyri*** conidia. ***V. pyri*** formed mainly dicellular conidia. The presence of 5 mM DPA caused ***V. pyri*** cell lysis, reducing the number of conidia. Scale bar = 10 μm.

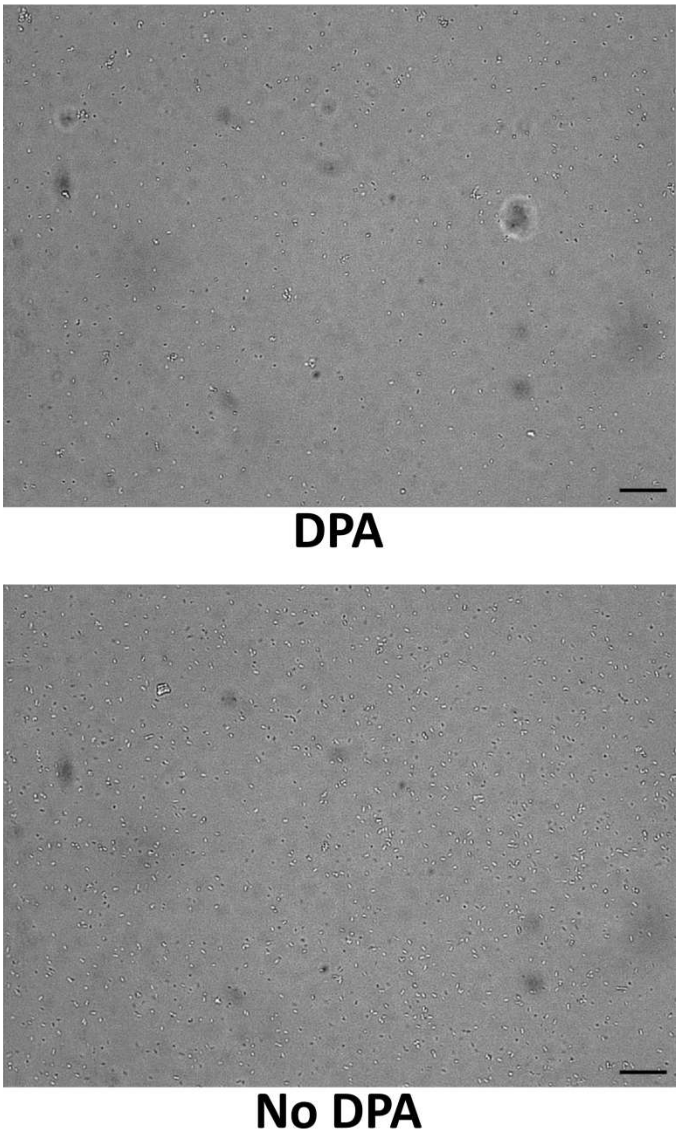

Figure S4. Number of conidia at pH 3 in the presence of 1 mM DPA. The control experiment was carried out in the absence of DPA. The images were collected with a Leica DM2500 microscope (x20 magnifications). Scale bar = 50 μm.

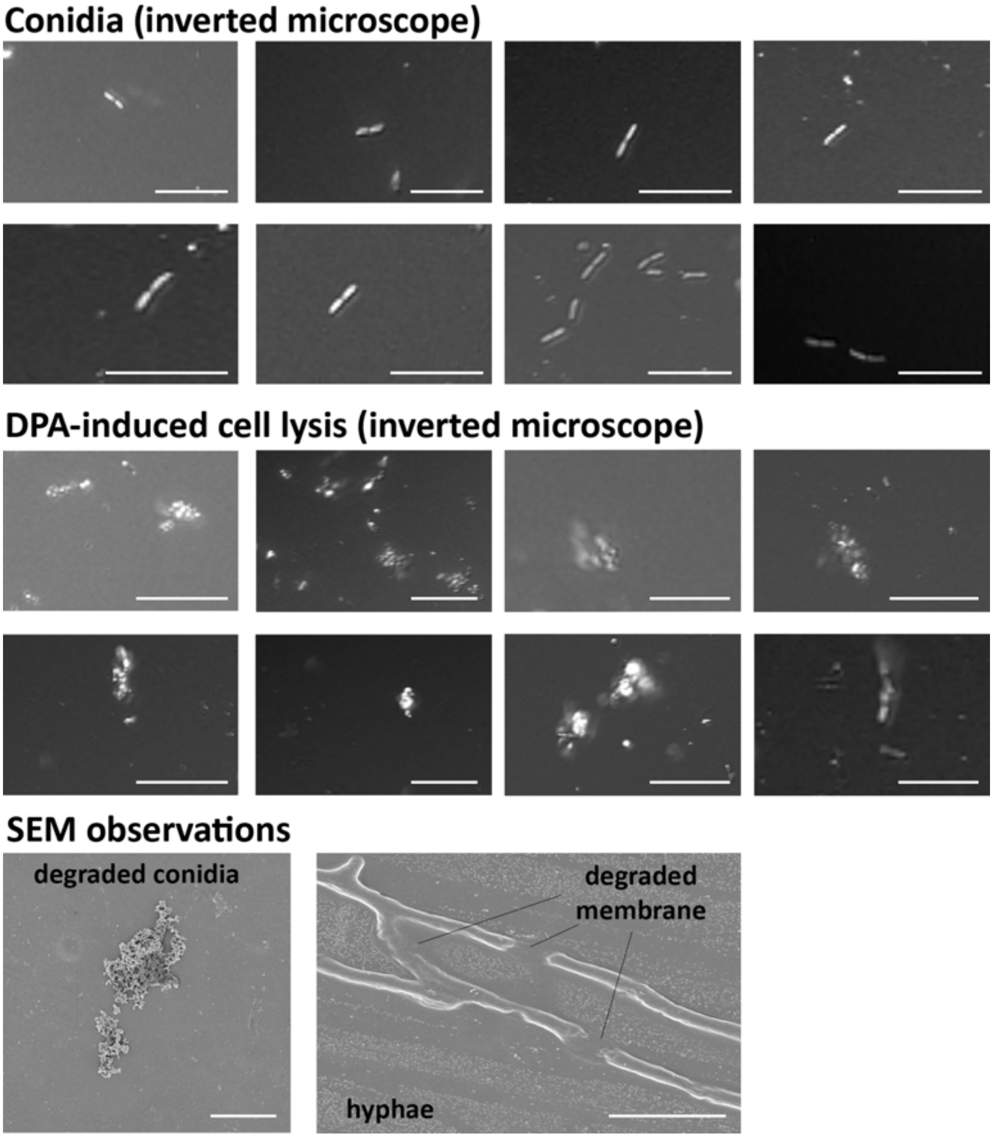

Figure S5. Observations of ***V. pyri*** cell lysis using SEM and inverted microscope. The degradation of the cell wall resulted in the formation of amorphous residues. Scale bar for inverted microscope = 10 μm. Scale bar for SEM = 2 μm.

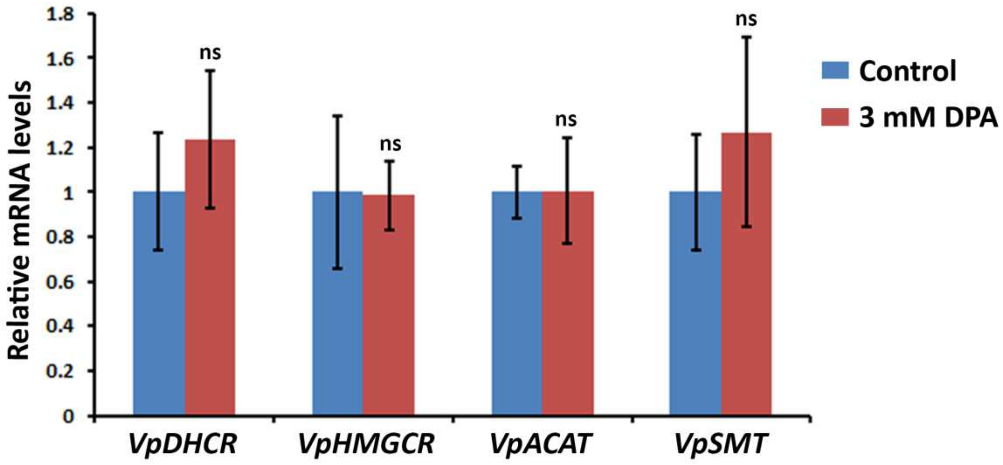

Figure S6. Relative mRNA levels of ergosterol biosynthetic pathway genes ***VpDHCR***, ***VpHMGCR***, ***VpACAT*** and ***VpSMT***. The obtained results indicated that DPA antifungal mechanism is not related to the biosynthesis of ergosterol.

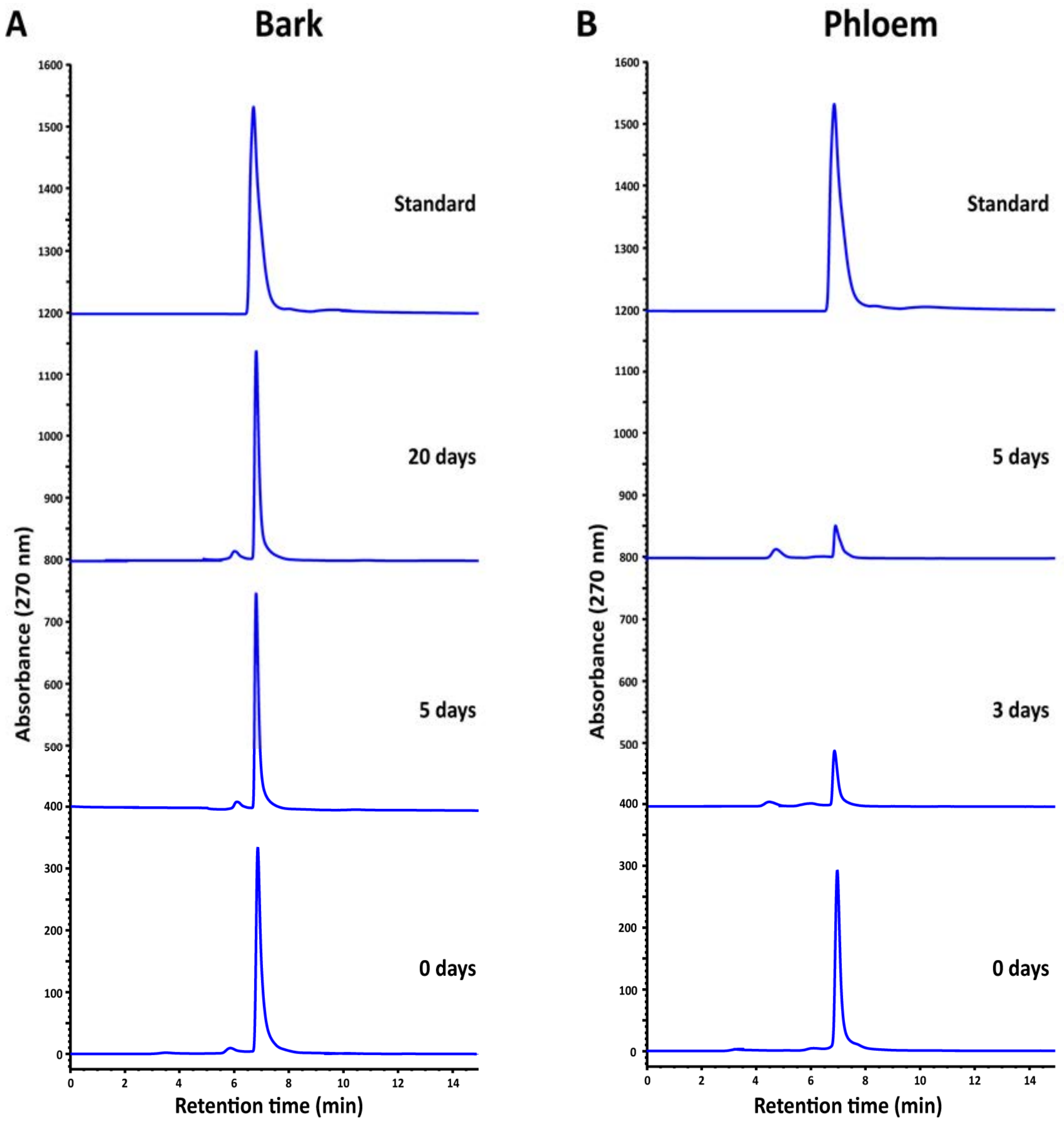

Figure S7. HPLC chromatograms of DPA in pear bark and phloem. DPA was detected in the phloem, demonstrating that DPA can cross the pear bark into the internal phases of the trunk. The concentration of DPA in the bark remained stable during 20 days, whereas significant DPA degradation was observed in the pear phloem after 3 days.

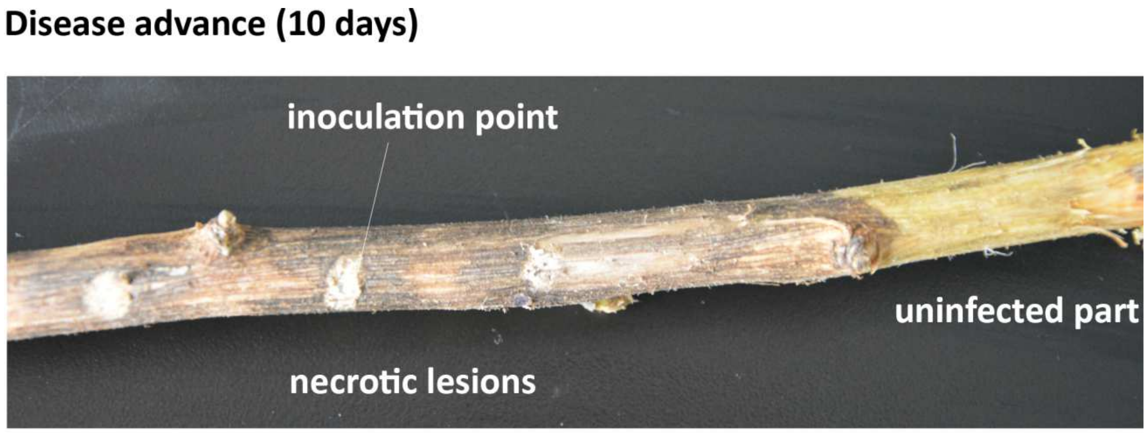

Figure S8. Lesions produced by ***V. pyri*** in pear trunks in the absence of DPA. The advance of the disease resulted in the death of the pear tree.
